# Antibiotic chemotherapy against heterogeneous pathogen populations in complex host tissues

**DOI:** 10.12688/f1000research.19441.1

**Published:** 2019-10-21

**Authors:** Dirk Bumann, Joseph Fanous, Jiagui Li, Frédéric Goormaghtigh

**Affiliations:** 1 Research Area Infection Biology, Biozentrum, University of Basel, Basel, CH-4056, Switzerland

**Keywords:** Antibiotics, Persistence, Heterogeneity

## Abstract

Antibiotic chemotherapy effectively cures many infections caused by susceptible bacterial pathogens. However, in some cases, even extended treatment duration does not completely eradicate the pathogenic bacteria from host tissues. A common model for underlying mechanisms assumes the stochastic formation of bacterial persisters similar to observations in laboratory cultures. However, alternative explanations related to the complexity of infected host tissues could also be relevant. We discuss several of these aspects and emphasize the need for integrated analysis as a basis for new control strategies.

Antibiotics have been saving the lives of millions of people. For most bacterial pathogens, short-term treatments of a few days effectively cure infections and prevent relapses ^1, 2^. However, in some cases such as tuberculosis and other chronic infections such as deep-seated abscesses with *Staphylococcus aureus*, severe typhoid fever, or polymicrobial infections of patients with cystic fibrosis, extended treatments over months and even years can fail to completely eradicate the pathogens from tissues, posing a risk for relapse ( Figure 1A). Many treatment failures are due to inheritable antibiotic resistance. However, surprisingly, treatment failures also occur when the pathogen retains full susceptibility to the antibiotics of choice in laboratory tests. There is an urgent medical need to improve the efficacy and shorten the treatments for these patients on the basis of a detailed mechanistic understanding of the problem. Several different factors influence antibiotic activities against pathogenic bacteria in host tissues. Most research groups active in the field focus on one particular factor: the stochastic variation of pathogen cells. However, other factors could be at least as important. In this review, we discuss some of these aspects and stress the need for an integrated analysis ( Figure 1B).

**Figure 1.**
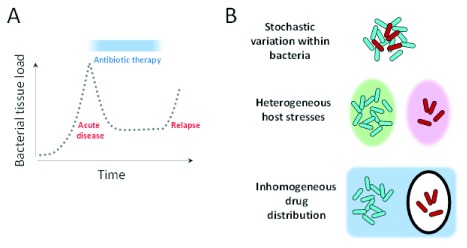
Treatment failures and potential causes. ( **A**) Incomplete eradication of bacterial pathogens during extended antimicrobial chemotherapy causes a risk for relapses after termination of therapy. ( **B**) Possible mechanisms that enable a bacterial subset (red) to survive during treatment while the rest of the bacterial population (blue) is successfully eradicated.

## Stochastic variation of bacterial properties in laboratory cultures

Bacterial cultures show heterogeneous properties even under completely homogeneous laboratory conditions indicating important endogenous stochastic variation within bacterial cells ^3– 5^. Exposure of bacterial cultures to lethal concentrations of bactericidal antibiotics rapidly kills most bacterial cells, but a small fraction of cells can survive for extended periods. It is possible that similar processes occur also in infected host tissues where they could contribute to incomplete eradication under antimicrobial chemotherapy. Because this phenomenon is readily observable *in vitro*, it has attracted the attention of a large number of research groups.

In many cases, the surviving small subset of bacteria represents non-growing remnant cells of a previous stationary culture that have an extended lag phase ^6^. Such persisters are thus one particular instance of the widely characterized extensive heterogeneity of stationary-phase cultures ^7^. A variety of other stress conditions also lead to increased antibiotic tolerance, including low ATP levels ^8^, over-expression of toxins or unrelated proteins ^9^, translation arrest ^10, 11^, oxidative stress ^12^, and pre-exposure of cells to sub-MIC levels of bactericidal antibiotics ^13^. Importantly, even growing bacteria show widely heterogeneous kill rates indicating that dormancy is not absolutely required for survival during antibiotic exposure ^14– 17^, implying alternative mechanisms leading to heterogeneous killing rates in clonal populations. These include asymmetric cell division ^4, 18, 19^ with uneven partitioning of efflux pumps among daughter cells ^20^, heterogeneous expression of prodrug-activating enzymes ^14^, transient gene amplifications ^21^, and heterogeneous induction of specific stress responses ^17^.

Although endogenous stochastic variation in bacteria often is assumed to play a major role in impairing antibiotic efficacy, empirical evidence is surprisingly scarce in infected tissues ^22^. Some clinical isolates of *Pseudomonas aeruginosa* and *Escherichia coli* from antibiotic-treated patients showed increased persister frequencies ^23, 24^, but this could also reflect fitness advantages of persisters under hostile host conditions or phage attacks. One argument against a general clinical relevance of persisters is the effectiveness of short-term antibiotic chemotherapy against many pathogens, which readily form refractory persisters in laboratory cultures ^1, 2^. Persisters thus might arise in patients but the host immune system seems to be capable of eradicating them quickly ^22^. Moreover, bacteriostatic antibiotics, which cause population-wide growth arrest in bacteria and make them tolerant against other antibiotics, are as effective as bactericidal antibiotics for the treatment of most infectious diseases ^25^. However, the distinction between bacteriostatic and bactericidal antibiotics is not absolute, as many “bacteriostatic” drugs can kill bacteria at higher exposure levels or during extended exposure times or both ^25^. In addition, translation arrest by bacteriostatic antibiotics might impair survival in host tissues due to an inability to produce essential virulence factors or stress defenses. Finally, non-growing persisters can also occur in biofilms where they could escape clearance by host phagocytes ^26^.

Although the general relevance of bacterial persisters in infectious diseases remains unclear ^27^, they might be crucial for infections with frequent relapses even after extended treatment durations ^28, 29^. However, other much less studied mechanisms could also contribute to infection relapse.

## Pathogen physiology in host tissues

Clinical microbiology relies largely on standard *in vitro* assays in rich media to assess antibiotic susceptibility of pathogens. The results often are predictive of therapeutic efficacy, but pathogen physiology is significantly different under assay conditions compared with infected host tissues ^30^. Genetic screens in diverse pathogens typically have revealed many hundreds of virulence genes that are specifically required in host tissues but not in rich broth cultures, indicating large-scale relevant functional differences. Several parameters that differ between tissues and broth cultures also have a major impact on antibiotic activities. This includes ^31^ oxygen tension ^32^, carbon dioxide tension ^33, 34^, metabolite concentrations ^35^, pH ^36, 37^, and antimicrobial effector molecules of the host immune system such as cationic antimicrobial peptides (CAMPs) ^38^ and nitric oxide ^39^. Limited nutrient supply and stress conditions can result in slow pathogen proliferation, which strongly affects the activity of most antibiotics ^40– 42^. Finally, pathogens can also adapt to the antibiotic exposure ^43^ and this adaptation might be more successful when antibiotics gradually penetrate into the infection site, compared with abrupt exposure in standard *in vitro* assays. All of these effects might lead to poor antibiotic efficacy, requiring extended treatment times.

## Pathogen heterogeneity in host tissues

As an additional complexity, pathogens show significantly increased single-cell heterogeneity in tissues and body fluids of human patients and infected animals, compared with homogeneous laboratory cultures ^3, 44– 46^. This includes wide variation in bacterial growth rates, aggregation state, drug-efflux pumps, metabolism, and stress responses. All of these parameters influence the activity of antimicrobials, and it is possible that pathogen subsets with favorable properties tolerate antimicrobial exposure much better than their conspecifics, making eradication more difficult. Host-induced pathogen heterogeneity can reflect inhomogeneous host microenvironments triggering differences in bacterial physiology ^45– 47^ but also host-induced pathogen activities that change local microenvironments ^48^.

An astonishing early finding was the differential recovery of *Mycobacterium tuberculosis* from open and closed (that is, no connection to airways) cavities in the lung of patients with tuberculosis ^49^. Whereas drug-resistant *M. tuberculosis* recovered from open cavities formed visible colonies on plates within a few weeks, *M. tuberculosis* from closed cavities of the same patients appeared only after a lag of many months. Strikingly, these colonies showed full drug susceptibility, suggesting limited selection for resistance development despite extended antimicrobial treatment. Sputum of patients with tuberculosis also contains mycobacteria with a wide range of cultivation phenotypes ^50^. A recent example that directly reveals heterogeneous bacteria physiology in tissues comes from *Salmonella* mouse infection models. *Salmonella* shows local and transient adaptations to divergent nutrient supply and disparate antimicrobial host attacks with reactive oxygen and nitrogen species ^51^. The resulting heterogeneity in *Salmonella* growth rates has a major impact on *Salmonella* eradication ^52– 54^.

## Drug concentration at the site of infection

Antimicrobials must reach bacterial cells to execute their bactericidal/bacteriostatic activities. Bacterial subsets hiding in tissue microenvironments with poor drug penetration might delay complete eradication during antimicrobial chemotherapy. Indeed, host anatomy and biochemistry might provide physical or chemical barriers (or both) for drug penetration. This includes the blood–brain barrier, bones, and serum proteins that bind antimicrobials, thereby decreasing their free concentration. Host inflammation increases tissue heterogeneity by altering endothelial permeability and the formation of lesions and abscesses. On the other hand, certain drugs such as fluoroquinolones, azithromycin, and bedaquiline accumulate in host phagocytes ^55– 57^, which might lead to elevated drug concentrations around the bacteria. Antimicrobial availability at the site of infection depends on the intestinal absorption of orally administered drugs, the distribution to the infected tissue, metabolism, and excretion. All of these processes depend on the physicochemical properties of the drug as well as the physiology of the patient, which might vary during bacterial infection. Drug penetration into accessible tissues can be measured by using microdialysis ^58^, but analysis of body fluids such as serum, cerebrospinal fluid, tracheal secretions, or urine is more common. It is unclear how representative these values are for drug availability around the bacterial cells during infection. Indeed, novel methods such as matrix-assisted laser desorption/ionization (MALDI) mass spectrometry imaging demonstrate remarkable differences in local drug concentrations in lesions compared with surrounding lung tissue in tuberculosis patients and animals infected with *M. tuberculosis* ^59, 60^. The emerging data suggest that inhomogeneous drug exposure could be a crucial factor for difficulties in eradicating *M. tuberculosis*. Unfortunately, experimental data are lacking for most other infectious diseases.

## Conclusions

Antibiotic chemotherapy of most bacterial infections is highly effective if the causative pathogen is susceptible to the antibiotic of choice. However, some infections require extended treatments to prevent relapses. Antibiotic-tolerant bacterial subsets (“persisters”) as observed in laboratory cultures might contribute to this problem. Alternatively, the host tissue environment could be decisive by providing inhomogeneous stress conditions and limiting drug distribution. To clarify these issues, we need more integrated *in vivo* research exploiting recent single-cell approaches and complementary techniques such as mass spectrometry imaging and three-dimensional high-resolution whole-organ microscopy ^61, 62^. A better understanding of the real problems impairing the chemotherapy of such infections is critically important to devise novel strategies for more effective and rapid treatments.
